# Effect of a 12-Week Almond-Enriched Diet on Biomarkers of Cognitive Performance, Mood, and Cardiometabolic Health in Older Overweight Adults

**DOI:** 10.3390/nu12041180

**Published:** 2020-04-23

**Authors:** Alison Mary Coates, Samantha Morgillo, Catherine Yandell, Andrew Scholey, Jonathan David Buckley, Kathryn Ann Dyer, Alison Marie Hill

**Affiliations:** 1 School of Health Sciences, University of South Australia, Adelaide 5001, SA, Australia; morsp008@mymail.unisa.edu.au (S.M.); catherine.yandell@unisa.edu.au (C.Y.); jon.buckley@unisa.edu.au (J.D.B.); kate.dyer@unisa.edu.au (K.A.D.); 2 Alliance for Research in Nutrition, Exercise and Activity (ARENA), University of South Australia, Adelaide 5001, SA, Australia; Alison.hill@unisa.edu.au; 3 Centre for Human Psychopharmacology, Swinburne University, Melbourne 3122, VIC, Australia; andrew@scholeylab.com; 4 School of Pharmacy and Medical Sciences, University of South Australia, Adelaide 5001, SA, Australia

**Keywords:** nuts, alertness, cardiometabolic health, overweight, lipids

## Abstract

Long term nut consumption is associated with reduced risk of coronary heart disease and better cognitive function. This study examined supplementing habitual diets with almonds or carbohydrate-rich snack foods (providing 15% energy) on biomarkers of cardiovascular and metabolic health, mood and cognitive performance. Participants (overweight/obese, 50–80 years) were randomised to an almond-enriched diet (AED) or isocaloric nut-free diet (NFD) for 12 weeks. Body weight, blood lipids, glucose, insulin, blood pressure (BP), arterial stiffness, cell adhesions molecules, C reactive protein (CRP), mood, and cognitive performance (working memory primary outcome), dietary profiles and energy intake/expenditure were measured at baseline and Week 12 in 128 participants (n = 63 AED, n = 65 NFD). Compared with NFD, AED was associated with altered macro and micronutrient profiles, but no differences in energy intake or expenditure. The AED significantly reduced triglycerides and SBP but there were no other changes in cardiometabolic biomarkers, mood, or cognitive performance. The inclusion of almonds in the diet improves aspects of cardiometabolic health without affecting cognitive performance or mood in overweight/obese adults.

## 1. Introduction

Habitual nut intake has long been associated with cardiovascular benefits including reduced risk of cardiovascular disease (CVD) [1], decreased incidence of metabolic syndrome (MetS) [2], and decreased risk of type II diabetes [3]. Tree nuts are an important source of nutrients, containing phytochemicals, antioxidants, and a healthy lipid profile (rich in mono and polyunsaturated fatty acids), all of which are thought to mediate the beneficial cardiovascular and metabolic effects through altered lipid metabolism, antioxidant, and anti-inflammatory mechanisms [4]. 

Components of MetS, e.g., hypertension, impaired glucose regulation, dyslipidemia, obesity, and inflammation represent modifiable risk factors for cognitive decline [5] and can impair cognitive function [6]. Improvement in these factors, through dietary change, has been shown to modulate cognitive performance [7]. Nut consumption represents one aspect of nutrition with wide ranging benefits that may counteract these negative influences, including reducing oxidative damage and inflammation, and improving vascular responsiveness [8,9,10]. Improvements in vascular function associated with nut intake are proposed to not only improve cardiovascular health but also contribute to improving cognitive function [11]. Clinical trials have found improvements in vascular health following consumption of walnuts [12], hazelnuts [13], and almonds [14], but this has not been a consistent finding in all studies, possibly due to differences in methods of assessing vascular health, differences in populations evaluated, dose, or length of the period of consumption [9,10]. 

Furthermore, there is now emerging literature to support nut consumption being associated with enhanced cognitive function or reduced cognitive decline [15,16]. Key nutrients found in nuts (and almonds in particular), including monounsaturated fat [17] and vitamin E [18] have been associated with reduced cognitive decline, and it has been proposed that regular nut consumption may prevent or slow the progressing of age-related brain dysfunction [16,19]. Prospective/longitudinal studies have demonstrated a positive association between nut consumption and cognitive performance [20,21,22] with a greater effect in those with the highest nut consumption [21]. In addition, prospective cohort studies reported improved cognitive performance and a reduced incidence of cognitive decline when the Mediterranean dietary pattern (with nuts as a key component) was adhered to [23,24].

There have been mixed results from randomized controlled trials assessing the effects of nut intake on cognition. A 12 week weight loss intervention including almonds found improvements in memory and attention but this was due to weight loss and not almonds, but almonds reduced the post-lunch dip in memory [25]. A cross-over study in University students consuming 60 g ground walnuts/d or a nut-free diet each for 8 weeks found no changes in memory, mood or non-verbal reasoning abilities but did find that walnuts may have the ability to increase inferential reasoning [26]. The WAHA study found no effect of global cognitive composite scores in a healthy older population (split across Spain and America) after 2 years of including ~15% energy from walnuts compared with a walnut-free diet. We have previously shown improvements in short-term memory, verbal fluency, and processing speed when middle-aged to older healthy adults consumed Hi-Oleic peanuts (56–84 g/day) for 12 weeks. There has been one pilot study with Brazil nuts in older adults with mild cognitive impairment showing an improvement in verbal fluency and constructional praxis after 6 months of supplementation with one Brazil nut per day [27].

Cross-sectional analyses of cohort studies have observed lower depression scores in habitual nut consumers [28,29], and one study showed that 8 weeks of walnut consumption in young healthy non-depressed adult males improved total mood disturbance [30]. It has been proposed that the nutrient profile of nuts may be neuroprotective, and a small number of intervention studies have compared the effects of consuming nuts on mood.

Co-monitoring cardiometabolic measures with mood and cognition may provide insight into the mechanisms underlying any changes in neurocognitive performance. The aim of this study was, therefore, to compare an almond-enriched diet with a nut-free diet over 12 weeks on biomarkers of cardiometabolic health, mood and cognitive performance in overweight/obese middle-aged to older adults.

## 2. Materials and Methods

### 2.1. Study Design

The study was a 12 week, two-arm, parallel-group randomised dietary intervention conducted at the University of South Australia Clinical Research Facility in Adelaide, Australia between January 2016 and September 2017. The study was approved by the University of South Australia Human Research Ethics Committee and registered with the Australian and New Zealand Clinical Trials Register (ACTRN12615001294549). The study was explained in detail and written informed consent was obtained from participants prior to commencing, and the study was conducted in accordance with the Declaration of Helsinki.

### 2.2. Participants

Adults aged 50–80 years (women postmenopausal), with body mass index (BMI) 25–39.9 kg/m^2^ were recruited in Adelaide, Australia through advertisements via newspapers, television, social media, medical centres, public noticeboards, and the University of South Australia website. Interested participants were screened for the following exclusion criteria: smoking, diagnosis of diabetes, liver, gastrointestinal or CV disease, uncontrolled hypertension (SBP/DBP > 160/100 mmHg), neurological disorders or history of inflammatory brain disease, history of depression or anxiety disorders in past 2 years, cognitive impairment or memory loss (score <18 on the Montreal Cognitive Assessment (MOCA) [31]), regular nut consumption (>30g per day), known hypersensitivity or allergy to nuts, were a restrained eater (score ≥12 on the three factor eating questionnaire [32]), pregnant or breastfeeding, unstable medications in the past 3 months, taking vitamin supplements, herbal extracts or illicit drugs.

### 2.3. Group Allocation and Blinding

Eligible participants were allocated to one of two groups containing either almonds (almond enriched diet, AED) or a nut-free diet (NFD) which provided energy-matched carbohydrate-rich snack foods. Treatment allocation was performed via minimisation [33] based on age; gender and BMI by an investigator who had no participant contact. All staff involved in data collection, analysis, and statistical analysis were blinded to treatment allocation until after completion of statistical analysis.

### 2.4. Study Intervention

#### 2.4.1. Study Foods

Whole natural raw almonds (Almond Board of California) were provided to those in the AED group. Participants were asked not to chop or grind them prior to consumption as this may influence the effects of almonds on outcome measures [34]. The NFD group were provided with carbohydrate-rich snack foods (The Original Scotch Finger, Arnott’s Biscuits, North Strathfield, Australia and No Added Salt Potato Chips, Freedom Foods, Taren Point, Australia) which were chosen as they were nut- and seed-free and could achieve the 15% energy intake with a manageable portion size. In addition, sweet biscuits and potato chips are a common snack in Australia as well as other countries and the same sweet biscuits have previously been used as a comparator food for almonds as a snack in Australia [35].

#### 2.4.2. Dietary Intervention

At the baseline visit, participants commenced their allocated diet under the supervision of a qualified dietitian and registered nutritionist. Individual estimated energy requirements (EER) were calculated using Harris-Benedict Equations based on sex, age, initial body weight and physical activity [36]. Participants were provided with a portion of snack foods equivalent to ~15% of their EER. For the AED group this was calculated to the nearest gram and for the NFD group this was to the nearest ½ biscuit (or grams for potato chips) for practical reasons. Participants were asked to consume their allocated snack food 6 days per week for 12 weeks.

All participants attended brief appointments every three weeks where they were weighed to check they were weight stable and to confirm that participants were consuming the snack foods (assessed via daily checklists and via multi-pass 24 h recalls) and to discuss strategies of how to incorporate the snack foods into their habitual diets. In both groups, participants were advised to substitute snack foods for discretionary foods, not to add foods to their usual diet. Participants were required to return any uneaten test foods so they could be weighed, and compliance assessed. Participants were provided with new test foods every three weeks to ensure freshness.

### 2.5. Procedure

Each clinic visit (Week 0 and Week 12) was conducted in the morning following an overnight fast (ab libitum water allowed) and 24 h free from alcohol and took approximately 2.5 h to complete. The same order of testing was followed for each participant on each occasion. If participants habitually took medications (stable dose) in the morning this was noted and the same procedure was followed on both testing occasions.

#### 2.5.1. Energy Intake and Dietary Assessment Via Quantitative Food Diaries

Participants were shown how to weigh/measure and record their dietary intake (digital scales were provided). Dietary intake was assessed based on the analysis of four days (1 weekend day) at the beginning and end of the dietary intervention. Analysis was performed using a computerised database (FoodWorks^®^ Professional Edition, version 8, 2012; Xyris Software, Highgate Hill, Australia) which was the most recent version at time of entry and energy, macro and micronutrient data was derived from the Australian Food and Nutrient (AUSNUT) 2011-13 food composition database [37]. Established cut offs of <4000 kJ or >17,000 kJ/day were used to exclude participants suspected of under- or over-estimating daily total energy intake [38].

#### 2.5.2. Estimated Energy Expenditure

During the intervention, participants were asked to maintain their normal physical activity patterns. Bouchard’s Physical Activity Record [39] was used to capture activity and derive energy expenditure. Data were collected in the week prior to baseline assessments and in the final week on the intervention, reported in 15-min intervals and categorised on a scale of increasing metabolic equivalents (1.0 to 7.8 METs) to give an estimate of daily energy expenditure in kilocalories (kcal/day).

### 2.6. Clinical Assessments

#### 2.6.1. Demographic and Morphometric Measures

Demographic data collected included age, gender, lipid and blood pressure medication usage, education, socio-economic status, race/ethnicity (Classification from Australian Standard Classification of Cultural & Ethnic Groups 2016), employment and marital status All anthropometric assessments were conducted with participants wearing light clothing and barefoot. Body mass and percentage body fat were measured using calibrated electronic scales (Tanita Ultimate Scale 2000; Tokyo, Japan). Measures of height and waist circumference were assessed as described previously [40]. Body mass index (BMI) was calculated using Quetelet’s index: mass (kg)/height (m)^2^ [41]. All assessments were conducted twice with the average value used in analyses.

#### 2.6.2. Cognitive Function and Mood Battery

Cognitive function was tested using the Computerised Mental Performance Assessment System (COMPASS) neuropsychological test battery (Northumbria University), which has previously been shown to be sensitive to nutritional interventions [42] Tasks were presented on a desktop PC and responses were completed using either a four-button response box or the computer mouse and keyboard depending on the task. For word recall, only pen-and-paper were used. The COMPASS battery was designed to measure the cognitive domains attention, episodic memory, and working memory using the following tasks: Immediate Word Recall, Delayed Word Recall, Word Recognition, Simple Reaction Time, Choice Reaction Time, Numeric Working Memory, Corsi Block, N-Back, Picture Recognition, Serial Subtraction (threes and sevens), Peg and ball, congruent and incongruent Stroop colour-words, and Rapid Visual Information Processing (RVIP) [43]. Participants were familiarised with the computerised cognitive test battery during screening to eliminate learning effects at the subsequent baseline visits and parallel versions of each task were used for the repeated testing sessions.

Composite measures for cognitive outcomes were also calculated. The tasks were grouped based on whether they represented a memory task (immediate and delayed word recall, numeric working memory, word recognition, picture recognition, serial threes and sevens) or an attention task (simple reaction time, choice reaction time, N-back, RVIP). These were then grouped based on whether they measured accuracy (% accuracy) or reaction time (ms).

This created six composite scores:

^1^Composite score for Attention-Reaction Time (RT) = (_z_Simple RT + _z_Choice RT + _z_Four Choice RT + _z_Rapid Visual Information Processing)/4
^2^Composite score for Attention-Accuracy = (_z_Four Choice RT + _z_Rapid Visual Information Processing + _z_Serial subtraction 3 + _z_Serial subtraction 7)/4
^3^Composite score for Working Memory-RT = (_z_Numeric Working Memory + _z_N-back + _z_Corsi blocks + _z_Rapid Visual Information Processing)/4
^4^Composite score for Working Memory-Accuracy = (_z_Numeric Working Memory + _z_N-back + _z_Corsi blocks + _z_Serial subtraction 3 + _z_Serial subtraction 7 + _z_Rapid Visual Information Processing)/6
^5^Composite score for Long Term Memory = (_z_Delayed Word Recall + zDelayed Word Recognition + _z_Delayed Picture Recognition + _z_Verbal Fluency + _z_Verbal Fluency Exclusion)/5
^6^Composite score for Executive Function = (_z_Stroop Congruent correct + _z_Stroop Incongruent correct + _z_Verbal Fluency + _z_Verbal Fluency Exclusion + _z_Serial subtraction 3 + _z_Serial subtraction 7 + _z_Peg and Ball (accuracy = negatively scored errors))/7

Mood state was assessed with a series of Visual Analogue Scales (VAS; a 100 mm horizontal line displayed on a computer monitor), with each endpoint labeled with antonyms (e.g., ‘muzzy’ and ‘clearheaded’; participants responded using a computer mouse cursor. Three dimensions of mood were assessed (alertness, calmness, and contentment) with 16 separate Bond-Lader VAS items [44]. and mood states were also evaluated using the Profile of Mood States (POMS) scale [45].

#### 2.6.3. Resting Blood Pressure and Arterial Compliance

Resting Systolic (SBP) and Diastolic blood pressure (DBP) and small (SAC) and large (LAC) arterial compliance were recorded using the Cardiovascular Profiler™ (HDI Cardiovascular Profiler CR2000, Hypertension Diagnostics, Eagan, MN, USA) after participants had been lying supine for 5–10 min with legs uncrossed [46]. Three consecutive readings were recorded at ~2 min intervals (with the average used for analysis) by automated oscillometry (blood pressure cuff placed over the left brachial artery) and by placing a tonometer over the right radial artery for blood pressure and pulse wave analysis, respectively.

#### 2.6.4. Biochemical Analyses

Fasting blood samples (10–12 h overnight) were obtained through venipuncture of a forearm vein using the Vacuette^®^ blood collection system. EDTA containing tubes were used to collect plasma for assessing triglycerides, total cholesterol, high-density lipoproteins (HDL-C) insulin, C-reactive protein, VCAM, ICAM, alpha tocopherol. Sodium Fluoride containing tubes were used to collect plasma for assessing glucose. Blood samples were centrifuged (4 °C, 4000 rpm, 10 min) before the plasma was aliquoted and frozen at −20 °C then stored at −80 °C.

Fasting plasma lipids (triglycerides, total cholesterol, HDL), glucose and high sensitivity C Reactive protein (CRP) concentrations were determined using a commercial assay kit with a Konelab 20XT clinical chemistry analyser (Thermo Fisher Scientific, Waltham, MA, USA). C Reactive Protein Values for CRP above 3 mg/L were excluded as they were considered to reflect low-grade inflammation [47].

Low density lipoprotein cholesterol (LDL-C) was calculated using the Friedewald equation [48]. Fasting plasma insulin concentrations were determined by ELISA (enzyme-linked immunosorbent assay) (Mercodia, Uppsala, Sweden). Reliability for these assays has been described previously [40]. The HOMA 2 online calculator was used to derive measures of insulin resistance (HOMA2-IR), insulin sensitivity (HOMA2-%S) and beta cell function (HOMA2-%B) from fasting glucose and insulin values [49].

Endothelial adhesion molecule concentrations of intercellular adhesion molecule 1 (ICAM 1) and vascular cell adhesion molecule 1 (VCAM-1) were measured by commercially available ELISA kits (Millipore Human Sepsis Panel 1 analysed on the Luminex MagPix^®^ plate reader). Plasma α-tocopherol levels were determined by reverse-phase high-performance liquid chromatography based on the method of Turner et al. [50].

### 2.7. Sample Size

The primary outcome measure for this trial was the composite score for working memory-reaction time. Power analysis indicated that a total of 68 participants per group were required to detect a difference in cognitive function between the two groups that equates to a medium effect size (0.5), at an α-level of 0.05 with a statistical power of 0.80. An additional 15 (~10%) were recruited to allow for attrition.

### 2.8. Statistical Analysis

Non–normally distributed variables were logarithmically transformed before analysis. Where normality was not achieved, nonparametric methods for analysis were used. Baseline characteristics between groups were assessed by independent student t-tests and chi-squared tests for continuous and categorical variables respectively. An intention to treat analysis was conducted as the main analysis. Changes over time (weeks 0–12) between the intervention groups were assessed using a linear mixed effects model with treatment as a between-subject factor, and time as the repeated measurement. Age, sex and BMI were controlled for in the mixed models where appropriate. In addition, sleepiness at the time of the cognitive testing was controlled for when assessing Bond-Lader mood scores. Where there was a significant main effect, Bonferroni post hoc comparisons were performed to determine differences between group means. The number of participants who completed the study with data available for each variable are shown in Table S1. Analyses were also conducted for participants who completed the study irrespective of compliance with data shown in Tables S2–S5. Statistical analyses were performed using SPSS version 25.0 (SPSS In., Chicago, IL, USA).

## 3. Results

### 3.1. Participants

**Figure 1:**
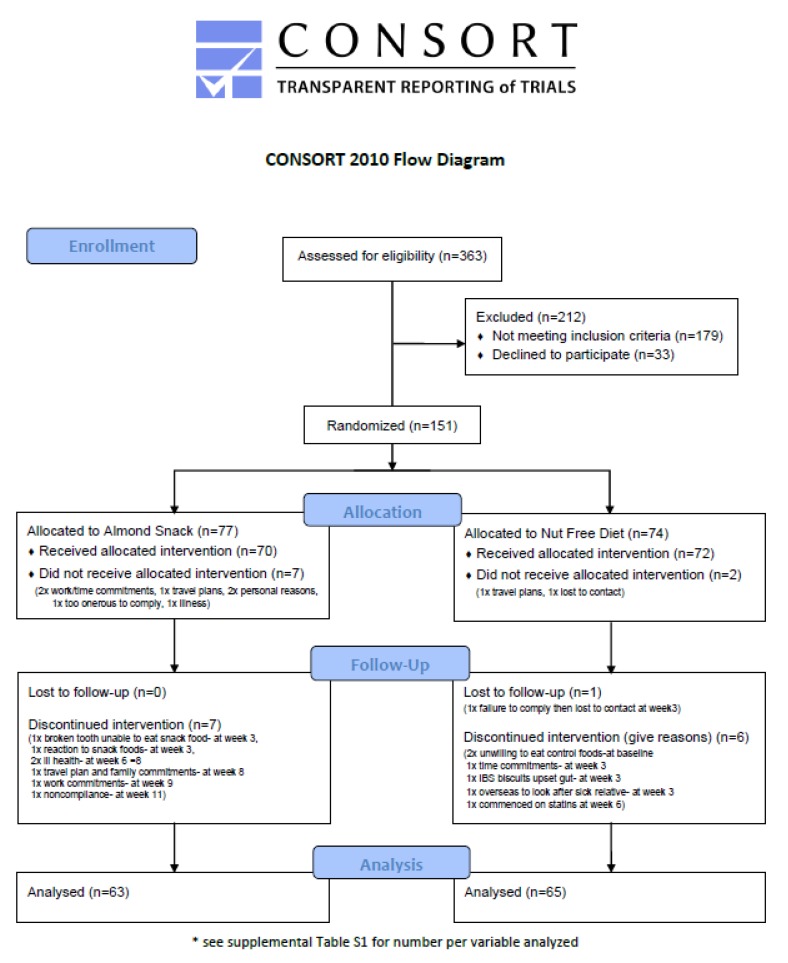
Consort diagram showing the flow of participants through the study.

A total of 363 participants were screened, 151 were randomised, 142 completed baseline and 128 (78 females, 70 males) completed the study (Figure 1). The demographics of participants are shown at baseline in Table 1 and there were no differences between those in the AED or NFD groups. Those who withdrew after baseline (n = 14) were heavier but did not differ in BMI or percent body fat and there were no differences in any measures of mood or cognition between the groups at baseline.

### 3.2. Nutrient Intake and Energy Balance

Overall energy intake did not differ between the AED and NFD groups (Table 2). However, there was a ∼20% greater fat intake, predominantly monounsaturated, and polyunsaturated fat and a small reduction in saturated fat. The AED group had a lower intake of energy from carbohydrate and the NFD had a lower intake of fibre (Table 2). Intakes of the following micronutrients were higher when almonds were consumed compared with the NFD: alpha-tocopherol, potassium, magnesium, calcium iron, and riboflavin but there were no significant differences in other nutrients There were no differences in total energy expenditure between the dietary phases (Table 2).

### 3.3. Cognition and Self Report Measures of Mood

Cognition and mood data are summarised in Table 3 and Table 4. There were no significant changes in any measures of individual tests of cognitive performance or in the composite scores for cognition. There was a trend for an improvement in alertness in the AED group compared with the NFD group, but no differences in other measures of mood.

### 3.4. Cardiometabolic Biomarkers

Cardiometabolic outcomes are summarised in Table 5. There were significant reductions in triglycerides and systolic blood pressure following the AED. There were no significant changes other cardiometabolic biomarkers, circulating alpha-tocopherol levels or body composition.

## 4. Discussion

Despite long term studies with regular nut consumption suggesting better cognitive performance [20], the present study failed to find significant changes in any measure of cognitive performance. It has been hypothesised that a regular nut intake may influence cognitive performance by improving systemic vasodilator function and enhancing vasodilator function in the cerebral arteries [11]. We found no significant difference in attention, memory or executive function between groups nor did we see changes in endothelial cell adhesion molecules or arterial stiffness, suggesting that this hypothesis may not be consistent for all nut types or the length of intervention or dose provided in this study was insufficient to elicit this change.

Almond consumption resulted in reductions in triglycerides compared with the NFD group. but there were no significant changes in total, LDL or HDL cholesterol. We did not see any changes in fasting glucose, insulin or insulin resistance (HOMA). These findings are somewhat in line with previous findings, with systematic reviews of randomised controlled trials with various nuts having reported improvements in MetS criteria [51]. Systematic reviews focused on almonds [52,53] or a range of tree nuts and peanuts have also found improvements in lipids [54] and insulin resistance (HOMA-IR) [55] and some studies have reported improvements in inflammatory biomarkers [56]. Furthermore, there was a significant reduction in systolic blood pressure in the AED group compared with the NFD group. A previous systematic review assessing the effects of nut consumption on blood pressure reported that only some studies found significant changes in blood pressure [57].

There were no significant changes in soluble endothelial adhesion molecules (ICAM or VCAM), nor changes in small or large arterial stiffness (inverse of arterial compliance used in this study). A previous study by our group in a similar population using the same measure of arterial compliance found a 10% reduction in small arterial compliance, but no change in LAC or blood pressure, following regular peanut consumption that provided 15% of total energy for 12 weeks [58]. A recent review comparing the effects of tree nut and peanut consumption on measures of vascular function excluding flow-mediated dilatation, found a lack of consistency in the literature which may reflect differences in techniques, population or ways nuts have been incorporated into the diet [10].

Animal and cell studies have explored other potential mechanisms of how nuts may improve cognitive performance and these studies have been summarised previously [16]. Proposed mechanisms include reduced damage to polyubiquitinated proteins (a hallmark of neurodegenerative disease) and upregulation of autophagy in the hippocampus which is involved in memory and cognitive performance; and the memory-improving activity of almonds may be attributed to their anticholinesterase, procholinergic, and cholesterol-reducing properties. It is important to consider that much of this evidence has come from animal models of ageing or dementia and Alzheimer’s disease and it is unclear how long these changes could take in humans or whether they would be observed at all.

The lack of change in measures of mood differs from findings in previous studies. Short term walnut consumption has been shown to improve mood in non-depressed healthy young men [30] and a short term cross-over intervention with a Mediterranean style diet containing nuts also reported improvements in alertness and contentment compared with habitual diet [59]. It has been suggested that improvements in mood may be associated with a higher consumption of magnesium [60] and whilst the AED resulted in a significant increase in magnesium intake this did not result in significant changes in alertness.

Over the 12 week intervention both groups remained weight stable and maintained their energy intake and total energy expenditure. However, we acknowledge that the Bouchard Diary likely overestimated energy expenditure as we have seen previously [61], despite it being a valid tool for capturing change in activity levels [62]. Consistent with the advice to substitute almonds for discretionary foods in the diet, the AED group had significant increase in monounsaturated fat and dietary alpha tocopherol, suggesting that participants were compliant with consuming almonds. While subtle changes in fibre intake were observed in both groups (1g/d increase AED; 3g/d decrease NFD), average fibre intake was consistent with Australian recommendations [63]. Interest in dietary fibre has increased due to its’ potential to modulate the gut microbiota [64], which may have cognitive effects ([65]). the quantity of almonds incorporated into the diet in the present study may not have been sufficient, or the substitutions that people made to incorporate almonds into their diet may have been too variable, to achieve positive effects on broader cardiometabolic biomarkers and cognitive performance.

Whilst the population in this study were free of diagnosed cardiovascular disease and diabetes, several required medications to control lipid and blood pressure, and the improvements in outcomes associated with the altered diet may have been masked as a result. Furthermore, while we selected people with BMIs in the overweight to obese range as elevated BMI has been associated with lower cognitive performance [66], the participants in this study did not have cognitive impairments and may have required more substantial dietary changes to see an effect, or the dietary changes in this study may have varied too much within the population to detect a significant group effect, or they may take longer to be observed. Whilst improvement in cerebrovascular and cognitive function have previously been demonstrated with peanuts [58], that study used a crossover design that accounted for between-people variation. The parallel study design used in the present study may have introduced additional variation, despite being adequately powered. In addition, the differences between the nutrient profile of almonds and peanuts may alter cognition differently.

## 5. Conclusions

In conclusion, this study failed to demonstrate, using a randomised controlled intervention trial study design, that inclusion of almonds in the diet of middle aged to older adults with a BMI in the range for overweight or obesity changed measures of cognition. It may be that a longer time-frame is necessary to see changes in cognitive performance, or that populations experiencing cognitive decline may be more sensitive to changes through dietary manipulation.

## Figures and Tables

**Table 1:** Baseline demographics of study population.

	All (n = 151)	Almond Diet (n = 77)	Nut-Free Diet (n = 74)
Age (years)	65 ± 8	64 ± 8	65 ± 8
Height (m)	1.67 ± 0.10	1.67 ± 0.09	1.67 ± 0.11
Weight (kg)	84.9 ± 13.0	84.4 ± 12.0	85.4 ± 14.0
BMI (kg/m^2^)	30.4 ± 3.7	30.3 ± 3.6	30.5 ± 3.8
WC (cm)	101.8 ± 10.5	101.2 ± 9.9	102.5 ± 11.0
Body fat (%)	35.6 ± 8.3	35.8 ± 8.5	35.3 ± 8.2
Ethnicity ^1^ (count)			
1 Oceanian	89	45	44
2 North-West European	28	12	16
3 Southern and Eastern European	12	8	4
4 North African and Middle Eastern	1	1	0
5 South-East Asian	2	1	1
6 North-East Asian	0	0	0
7 Southern and Central Asian	1	1	0
8 Peoples of the Americas	5	1	4
9 Sub-Saharan African	2	1	1
Medications			
% of population takingCholesterol lowering medication	18.4	14.3	22.5
Blood pressure lowering medication	31.9	25.7	38.0

Data presented as Mean ± SD, count, and percentage of population. ^1^ Ethnicity defined as per the Classification from Australian Standard Classification of Cultural & Ethnic Groups 2016.

**Table 2:** Effect of treatment on dietary nutrient intake and energy expenditure as determined weighed food diaries and activity diaries.

	Almond Diet		Nut-Free Diet		*P* Value	*P* Value	*P* Value
	Pre-Treatment	Post-Treatment	Pre-Treatment	Post-Treatment	Group	Time	Group × Time
Energy (kJ)	9118 ± 647	9624 ± 659	8975 ± 644	8782 ± 659	0.133	0.385	0.053
Carbohydrate (g)	228 ± 24	211 ± 24	227 ± 23	226 ± 24	0.456	0.086	0.153
Carbohydrate (%en)	41 ± 3	36 ± 3	41 ± 3	42 ± 3	0.001	<0.0001	<0.0001
Sugars (g)	109 ± 5	99 ± 5	110 ± 5	93 ± 5	0.679	<0.0001	0.300
Protein (g)	95 ± 3	99 ± 3	94 ± 3	88 ± 3	0.125	0.670	0.008
Protein (%en)	18 ± 5	18 ± 5	18 ± 5	17 ± 5	0.706	0.029	0.163
Fat (g)	87 ± 9	106 ± 9	82 ± 9	80 ± 9	<0.0001	0.001	<0.0001
Fat (%en)	35 ± 1	41 ± 1	34 ± 1	34 ± 1	<0.0001	<0.0001	<0.0001
Monounsaturated fat (g)	32 ± 12	47 ± 12	31 ± 12	29 ± 12	<0.0001	<0.0001	<0.0001
Monounsaturated fat (%en)	13 ± 2	19 ± 2	13 ± 2	12 ± 2	<0.0001	<0.0001	<0.0001
Polyunsaturated fat (g)	13 ± 5	19 ± 5	12 ± 5	10 ± 5	<0.0001	<0.0001	<0.0001
Polyunsaturated fat (%en)	5 ± 0	7 ± 0	5 ± 0	4 ± 0	<0.0001	<0.0001	<0.0001
Saturated fat (g)	34 ± 1	31 ± 1	32 ± 1	34 ± 1	0.885	0.828	0.050
Saturated fat (%en)	14 ± 1	12 ± 1	13 ± 1	14 ± 1	0.038	0.620	<0.0001
Alcohol (g)	8 ± 2	8 ± 2	10 ± 2	11 ± 2	0.258	0.646	0392
Fibre (g)	28 ± 1	29 ± 1	27 ± 1	24 ± 1	0.030	0.564	0.002
Alpha tocopherol (mg)	12 ± 4	26 ± 4	11 ± 4	10 ± 4	<0.0001	<0.0001	<0.0001
Sodium (mg)	2451 ± 113	2397 ± 145	2498 ± 110	2687 ± 143	0.230	0.563	0.298
Potassium (mg)	3463 ± 117	3601 ± 122	3451 ± 116	3182 ± 121	0.120	0.378	0.007
Magnesium (mg)	397 ± 12	487 ± 13	389 ± 12	330 ± 12	<0.0001	0.071	<0.0001
Calcium (mg)	1065 ± 68	1094 ± 67	1020 ± 67	911 ± 67	0.057	0.208	0.031
Iron (mg)	12 ± 0	14 ± 1	13 ± 0	11 ± 1	0.248	0.620	<0.0001
Niacin (mg)	25 ± 6	25 ± 6	25 ± 6	23 ± 6	0.530	0.341	0.355
Niacin Equivalents (mg)	43 ± 3	44 ± 3	43 ± 3	40 ± 3	0.205	0.475	0.099
Riboflavin (mg)	2.2 ± 0.1	2.9 ± 0.1	2.3 ± 0.1	2.0 ± 0.1	0.002	0.025	<0.0001
Caffeine (mg)	222 ± 18	209 ± 14	204 ± 17	195 ± 14	0.393	0.414	0.854
Energy Expenditure (kJ)	14463 ± 270	14505 ± 266	14694 ± 280	14280 ± 273	0.580	0.989	0.234

Estimated Marginal Means ± SEM presented from Linear Mixed Model, at baseline (pre-treatment) and 12weeks (post-treatment). Statistical significance *p* < 0.05.

**Table 3:** Effect of treatment on cognition.

	Almond Diet		Nut-Free Diet				
	Pre-Treatment	Post-Treatment	Pre-Treatment	Post-Treatment	Group (*p*)	Time (*p)*	Group × Time Interaction (*p)*
Attention RT (composite Z-score)^1^	−0.006 ± 0.081	−0.006 ± 0.079	0.019 ± 0.081	−0.067 ± 0.078	0.861	0.340	0.347
Attention Accuracy (composite Z-score)^2^	−0.087 ± 0.067	−0.040 ± 0.071	0.064 ± 0.066	0.036 ± 0.070	0.217	0.769	0.264
Working Memory RT (composite Z-score)^3^	0.030 ± 0.065	−0.030 ±0.070	0.027 ± 0.066	−0.065 ± 0.069	0.818	0.094	0.725
Working Memory Accuracy (composite Z-score)^4^	−0.122 ± 0.080	−0.065 ± 0.087	0.090 ± 0.077	0.061 ± 0.085	0.138	0.627	0.148
Long Term Memory Accuracy (composite Z-score)^5^	−0.079 ± 0.066	0.020 ± 0.074	−0.082 ± 0.065	0.100 ± 0.073	0.672	0.001	0.318
Executive Function Accuracy (composite Z-score)^6^	−0.151 ± 0.069	−0.078 ± 0.075	0.074 ± 0.067	0.120 ± 0.074	0.028	0.084	0.693
Simple RT (ms)	345 ± 13	350 ± 12	354 ± 12	346 ± 12	0.861	0.836	0.523
Choice RT -correct responses (%)	94.82 ± 0.71	94.84 ± 0.58	94.43 ± 0.70	94.78 ± 0.57	0.771	0.707	0.741
Choice RT (ms)	490 ± 10	502 ± 11	495 ± 10	485 ± 11	0.611	0.919	0.161
Four choice RT- correct responses (%)	99.64 ± 0.10	99.72 ± 0.13	99.75 ± 0.10	99.50 ± 0.13	0.688	0.392	0.085
Four choice RT (ms)	690 ± 15	681 ± 16	713 ± 15	678 ± 16	0.624	0.008	0.108
Rapid visual information processing -correct responses (%)	33.1 ± 2.4	36.4 ± 2.3	35.1 ± 2.3	38.2 ± 2.6	0.560	0.015	0.924
Rapid visual information processing RT (ms)	539 ± 8	537 ± 8	540 ± 8	540 ± 8	0.809	0.823	0.795
Rapid visual information processing (false alarms)	10.1 ± 1.8	11.8 ± 1.8	12.6 ± 1.8	12.9 ± 1.8	0.459	0.182	0.363
Numeric working memory accuracy (%)	89.16 ± 1.12	89.70 ± 1.17	91.86 ± 1.11	92.65 ± 1.16	0.056	0.330	0.861
Numeric working memory RT (ms)	1301 ± 39	1291 ± 39	1245 ± 39	1183 ± 39	0.121	0.047	0.155
N-Back correct Reponses (%)	73.59 ± 2.49	73.60 ± 2.87	75.83 ± 2.46	74.09 ± 2.82	0.791	0.845	0.844
N-Back RT (ms)	868 ± 33	890 ± 39	912 ± 32	886 ± 38	0.640	0.950	0.361
Corsi Blocks -span (score)	5.07 ± 0.12	5.16 ± 0.13	5.23 ± 0.11	5.04 ± 0.13	0.889	0.551	0.132
Corsi Blocks RT (ms)	8320 ± 344	7722 ± 293	8670 ± 340	8257 ± 289	0.242	0.042	0.707
Peg and Ball planning time (before responding) (ms)	6399 ± 293	6032 ± 279	6098 ± 289	5669 ± 275	0.368	0.017	0.852
Peg and Ball execution time (ms)	16918 ± 614	15943 ± 546	16045 ± 607	14671 ± 539	0.169	0.000	0.444
Peg and Ball Errors (score)	4.66 ± 0.46	4.02 ± 0.48	4.02 ± 0.45	3.16 ± 0.47	0.141	0.074	0.786
Immediate Word Recall- correct responses(score)	5.36 ± 0.24	5.61 ± 0.25	5.55 ± 0.24	6.38 ± 0.24	0.105	0.002	0.094
Delayed Word Recall- correct responses (score)	3.61 ± 0.24	4.02 ± 0.26	3.88 ± 0.24	4.30 ± 0.26	0.383	0.019	0.983
Word Recognition- correct responses (%)	80.26 ± 1.17	81.71 ± 1.12	77.43 ± 1.15	81.44 ± 1.10	0.253	0.002	0.147
Word Recognition RT (ms)	1265 ± 38	1277 ± 42	1330 ± 38	1348 ± 42	0.177	0.560	0.917
Picture Recognition- correct responses (%)	96.94 ± 0.51	96.46 ± 0.52	96.05 ± 0.50	96.52 ± 0.51	0.518	0.975	0.170
Picture Recognition RT (ms)	957 ± 26	946 ± 26	1000 ± 26	991 ± 26	0.210	0.442	0.966
Stroop Congruent accuracy (%)	99.08 ± 0.31	99.02 ± 0.29	99.22 ± 0.30	98.81 ± 0.28	0.915	0.395	0.515
Stroop Incongruent accuracy (%)	95.08 ± 0.89	95.26 ± 0.91	97.23 ± 0.87	97.80 ± 0.90	0.041	0.496	0.729
Stroop Congruent RT (ms)	1163 ± 38	1115 ± 37	1130 ± 37	1084 ± 36	0.492	0.063	0.957
Stroop Incongruent RT (ms)	1245 ± 42	1262 ± 41	1262 ± 41	1230 ± 40	0.905	0.700	0.290
Serial 3 (number of responses)	20.7 ± 1.3	20.4 ± 1.3	23.7 ± 1.2	23.7 ± 1.3	0.071	0.813	0.772
Serial 3 accuracy (%)	18.27 ± 1.29	17.71 ± 1.36	21.39 ± 1.27	20.72 ± 1.35	0.089	0.248	0.917
Serial 7 (number of responses)	14.8 ± 1.1	15.3 ± 1.2	18.3 ± 1.1	18.1 ± 1.1	0.041	0.653	0.333
Serial 7 accuracy (%)	11.46 ± 1.13	12.01 ± 1.20	15.13 ± 1.11	14.72 ± 1.19	0.046	0.875	0.279

Estimated Marginal Means ± SEM presented from Linear Mixed Model (including age, gender and BMI as covariates), at baseline (pre-treatment) and 12weeks (post-treatment). Statistical significance *p* < 0.05. RT = reaction time, ms = milliseconds. ^1^Composite score for Attention -Reaction Time (RT) = (_z_Simple RT + _z_Choice RT + _z_Four Choice RT + _z_Rapid Visual Information Processing)/4. ^2^Composite score for Attention -Accuracy = (_z_Choice RT + _z_Four Choice RT + _z_Rapid Visual Information Processing + _z_Serial subtraction 3 + _z_Serial subtraction 7)/5. ^3^Composite score for Working Memory-RT = (_z_Numeric Working Memory + _z_N-back + _z_Corsi blocks + _z_Rapid Visual Information Processing)/4. ^4^Composite score for Working Memory -Accuracy = (_z_Numeric Working Memory + _z_N-back + _z_Corsi blocks + _z_Serial subtraction 3 + _z_Serial subtraction 7 + _z_Rapid Visual Information Processing)/6. ^5^Composite score for Long Term Memory = (_z_Delayed Word Recall + zDelayed Word Recognition + _z_Delayed Picture Recognition + _z_Verbal Fluency + _z_Verbal Fluency Exclusion)/5. ^6^Composite score for Executive Function = (_z_Stroop Congruent correct + _z_Stroop Incongruent correct + _z_Verbal Fluency + _z_Verbal Fluency Exclusion + _z_Serial subtraction 3 + _z_Serial subtraction 7 + _z_Peg and Ball (accuracy = negatively scored errors))/7.

**Table 4:** Effect of Treatment on mood.

	Almond Diet		Nut-Free Diet				
	Pre-Treatment	Post-Treatment	Pre-Treatment	Post-Treatment	Group (*p*)	Time (*p)*	Group × Time Interaction (*p)*
*Profile of Mood States*							
Total mood disturbance	0.89 ± 1.90	1.11 ± 2.20	−3.74 ± 1.88	−2.22 ± 2.17	0.117	0.538	0.644
Tension	4.63 ± 0.53	4.28 ± 0.43	3.93 ± 0.52	3.56 ± 0.43	0.222	0.305	0.991
Depression	2.04 ± 0.41	2.43 ± 0.51	1.23 ± 0.41	1.45 ± 0.50	0.093	0.413	0.827
Anger	1.36 ± 0.34	1.00 ± 0.33	1.02 ± 0.33	1.02 ± 0.33	0.663	0.536	0.543
Fatigue	4.35 ± 0.43	4.86 ± 0.57	3.38 ± 0.43	3.72 ± 0.56	0.079	0.268	0.826
Confusion	5.21 ± 0.40	5.09 ± 0.42	4.35 ± 0.40	4.14 ± 0.41	0.078	0.543	0.869
Vigour	16.70 ± 0.70	16.40 ± 0.77	17.68 ± 0.70	16.06 ± 0.76	0.733	0.042	0.163
*Bond-Lader Visual Analogue Scale*							
Alert	55.92 ± 1.88	59.67 ± 1.99	61.37 ± 1.86	61.00 ± 1.96	0.173	0.133	0.067
Content	67.62 ± 1.78	70.38 ± 2.01	71.81 ± 1.77	71.74 ± 1.98	0.255	0.233	0.207
Calm	51.71 ± 2.22	55.12 ± 2.13	54.54 ± 2.20	57.34 ± 2.10	0.353	0.031	0.832
*Karolinska Sleepiness Score*	4.13 ± 0.18	4.18 ± 0.19	4.06 ± 0.18	4.14 ± 0.19	0.809	0.531	0.892

Estimated Marginal Means ± SEM presented from Linear Mixed Model, at baseline (pre-treatment) and 12weeks (post-treatment). Statistical significance *p* < 0.05. Statistical model for Profile of Mood States variables included age, gender, body mass index (BMI) and Bond-Lader Alertness as covariates. Statistical model for Bond-Lader variables included age, gender, BMI and Karolinska Sleepiness score as covariates. Statistical model for Karolinska Sleepiness Score included age, gender and BMI.

**Table 5:** Effect of Treatment on Cardiometabolic Parameters.

	Almond Diet		Nut-Free Diet				
	Pre-Treatment	Post-Treatment	Pre-Treatment	Post-Treatment	Group (*p*)	Time (*p)*	Group × Time Interaction (*p)*
Total cholesterol (mmol/L)	5.10 ± 0.13	4.91 ± 0.11	5.25 ± 0.12	5.21 ± 0.11	0.150	0.031	0.159
HDL cholesterol (mmol/L)	1.23 ± 0.04	1.44 ± 0.04	1.46 ± 0.04	1.47 ± 0.04	0.571	0.302	0.993
LDL cholesterol (mmol/L)	3.08 ± 0.12	2.94 ± 0.10	3.27 ± 0.11	3.19 ± 0.10	0.130	0.027	0.491
Triglycerides (mmol/L)	1.29 ± 0.07	1.15 ± 0.06	1.16 ± 0.07	1.18 ± 0.06	0.548	0.078	0.008
Ratio Total chol:HDL	3.86 ± 0.14	3.58 ± 0.12	3.78 ± 0.14	3.67 ± 0.12	0.969	0.000	0.102
Systolic Blood Pressure (mm/Hg)	133 ± 1	128 ± 2	132 ± 1	131 ± 2	0.550	0.002	0.044
Diastolic Blood Pressure (mm/Hg)	77 ± 1	75 ± 1	76 ± 1	76 ± 1	0.965	0.018	0.148
Glucose (mmol/L)	5.6 ± 0.1	5.6 ± 0.1	5.6 ± 0.1	5.5 ± 0.1	0.958	0.388	0.292
Insulin (mU)	7.17 ± 0.48	7.42 ± 0.48	7.51 ± 0.47	7.18 ± 0.47	0.934	0.859	0.213
Small Arterial Compliance (ml/mmHg × 100)	5.2 ± 0.3	5.3 ± 0.4	4.9 ± 0.3	5.2 ± 0.4	0.699	0.312	0.711
Large Arterial Compliance (ml/mmHg × 10)	15.2 ± 0.4	16.2 ± 0.5	16.2 ± 0.4	16.4 ± 0.5	0.335	0.070	0.227
Intracellular Adhesion Molecule (ng)	172 ± 10	188 ± 19	170 ± 10	154 ± 19	0.327	0.983	0.162
Vascular Cell Adhesion Molecule (ng)	1123 ± 29	1148 ± 32	1056 ± 29	1050 ± 31	0.057	0.400	0.228
HOMA2-IR	0.96 ± 0.06	0.99 ± 0.06	1.0 ± 0.06	0.95 ± 0.06	0.963	0.830	0.191
HOMA2-%B	73.5 ± 3.5	74.0 ± 3.3	72.0 ± 3.4	71.7 ± 3.2	0.666	0.936	0.813
HOMA2-%S	134.4 ± 9.0	132.7 ± 8.1	139.1 ± 8.8	136.5 ± 8.0	0.694	0.680	0.930
Alpha tocopherol (µg/mL)	4.85 ± 0.26	4.86 ± 0.21	4.69 ± 0.25	4.53 ± 0.21	0.435	0.526	0.481
logCRP	0.33 ± 0.051	0.344 ± 0.057	0.352 ± 0.050	0.300 ± 0.056	0.919	0.639	0.257
Weight (kg) *	84.52 ± 1.40	84.80 ± 1.38	85.15 ± 1.38	85.06 ± 1.36	0.819	0.418	0.141
BMI (kg/m^2^) *	30.23 ± 0.44	30.45 ± 0.44	30.56 ± 0.43	30.38 ± 0.43	0.791	0.297	0.125
Waist Circumference (cm) *	101.2 ± 1.1	101.4 ± 1.1	102.3 ± 1.1	102.0 ± 1.1	0.594	0.738	0.311
Body fat (%) *	35.8 ± 0.6	35.7 ± 0.6	35.5 ± 0.6	35.4 ± 0.6	0.747	0.464	0.839

Estimated Marginal Means ± SEM presented from Linear Mixed Model (including age, gender and BMI as covariates (*statistical model does not include BMI as covariate)), at baseline (pre-treatment) and 12weeks (post-treatment). Statistical significance *p* < 0.05. CRP was logged transformed to account for non-normally distributed data.

## Supplementary Materials

The following are available online at https://www.mdpi.com/2072-6643/12/4/1180/s1. Table S1: Number of participants with available data for each outcome variable in the Completers analysis; Table S2: Effect of treatment on dietary nutrient intake and energy expenditure as determined weighed food diaries and activity diaries. Completers analysis, Table S3: Effect of Treatment on cognition. Completers analysis, Table S4: Effect of Treatment on mood. Completers analysis, Table S5 Effect of Treatment on Cardiometabolic Parameters. Completers analysis.
