# Supplementary material for: Effect of a 12-Week Almond-Enriched Diet on Biomarkers of Cognitive Performance, Mood, and Cardiometabolic Health in Older Overweight Adults
Source: Nutrients. 2020 Apr 23;12(4):1180. doi: 10.3390/nu12041180 (PMC7230374; doi:10.3390/nu12041180)
Supplement: Supplementary file 1 [file nutrients-12-01180-s001.pdf]

Supplemental Table S1- number of participants included in the analysis for each variable in the Completers analysis

	Almond Diet		Nut-Free Diet	
	Pre-treatment	Post-treatment	Pre-treatment	Post-treatment
**Total Completers for analysis (n=128)**	63	63	65	65
Attention RT (composite Z-score)[1]	59	59	59	62
Attention Accuracy (composite Z-score)[2]	62	62	65	65
Working Memory RT (composite Z-score)[3]	62	62	60	63
Working Memory Accuracy (composite Z-score)[4]	58	58	65	60
Long Term memory Accuracy (composite Z-score)[5]	63	63	65	65
Executive Function Accuracy (composite Z-score)[6]	60	60	65	65
Simple RT (ms)	60	60	65	63
Choice RT (ms)	63	63	63	65
Four choice RT- correct responses (%)	63	63	65	65
Four choice RT (ms)	63	63	65	65
Rapid visual information processing – correct responses (%)	63	63	65	65
Rapid visual information processing RT (ms)	62	62	61	63
Rapid visual information processing (false alarms)	62	62	65	64
Numeric working memory accuracy (%)	63	63	65	65
Numeric working memory RT (ms)	63	63	64	65
N-Back correct Reponses (%)	63	63	65	65
N-Back RT (ms)	63	63	65	65
Corsi Blocks -span (score)	59	59	65	60
Corsi Blocks RT (ms)	63	63	65	65
Peg and Ball planning time (before responding) (ms)	63	63	65	65
Peg and Ball execution time (ms)	63	63	64	65
Peg and Ball Errors (score)	63	63	65	65
Immediate Word Recall- correct responses(score)	63	63	64	65
Delayed Word Recall- correct responses (score)	63	63	65	65
Word Recognition- correct responses (%)	63	63	65	65
Word Recognition RT (ms)	63	63	64	64
Picture Recognition- correct responses (%)	63	63	65	65
Picture Recognition RT (ms)	63	63	65	65
Stroop Congruent accuracy (%)	63	63	65	65
Stroop Incongruent accuracy (%)	61	61	65	65
Stroop Congruent RT (ms)	62	62	65	65
Stroop Incongruent RT (ms)	62	62	65	65

Serial 3 Total (number of responses)	62	23	65	65
Serial 3 accuracy (%)	62	62	65	65
Serial 7 Total (number of responses)	62	62	65	65
Serial 7 accuracy (%)	62	62	65	65
Profile of Mood States				
Total mood disturbance	63	63	65	65
Tension	63	63	65	65
Depression	63	63	65	65
Anger	63	63	65	65
Fatigue	63	63	65	65
Confusion	63	63	65	65
Vigour	63	63	65	65
Bond-Lader Visual Analogue Scale				
Alert	63	63	65	65
Content	63	63	65	65
Calm	63	63	65	65
Karolinska Sleepiness Score	63	63	65	65
Total cholesterol (mmol/L)	61	61	65	62
HDL cholesterol (mmol/L)	61	61	65	62
LDL cholesterol (mmol/L)	61	61	65	62
Triglycerides (mmol/L)	61	61	65	62
Ratio Total chol:HDL	61	61	65	62
Systolic Blood Pressure (mm/Hg)	63	63	65	64
Diastolic Blood Pressure (mm/Hg)	63	63	65	64
Glucose (mmol/L)	61	61	65	62
Insulin (mU)	62	62	65	62
Small Arterial Compliance (ml/mmHg x 100)	61	61	65	63
Large Arterial Compliance (ml/mmHg x 10))	60	60	65	63
Intracellular Adhesion Molecule (ng)	62	62	65	62
Vascular Cell Adhesion Molecule (ng)	62	62	65	62
HOMA2-IR	62	62	65	62
HOMA2-%B	62	62	65	62
HOMA2-%S	62	62	65	62
Plasma alpha tocopherol (μg/mL)	61	62	65	63
logCRP	39	39	38	38
Weight (kg)	63	63	65	65

BMI (kg/m^2)	63	63	64	65
Waist Circumference (cm)	62	62	65	65
Body fat (%)	63	63	65	65
Energy (kJ)	61	61	65	63
Carbohydrate (g)	61	61	65	63
Carbohydrate (%en)	61	61	65	63
Sugars (g)	61	61	65	63
Protein (g)	61	61	65	63
Protein (%en)	61	61	65	63
Fat (g)	61	61	65	63
Fat (%en)	61	61	65	63
Monounsaturated fat (g)	61	61	65	63
Monounsaturated fat (%en)	61	61	65	63
Polyunsaturated fat (g)	61	61	65	63
Polyunsaturated fat (%en)	61	61	65	63
Saturated fat (g)	61	61	65	63
Saturated fat (%en)	61	61	65	63
Alcohol (g)	61	61	65	63
Fibre (g)	61	61	65	63
Alpha tocopherol (mg)	61	61	65	63
Sodium (mg)	61	61	65	63
Potassium (mg)	61	61	65	63
Magnesium (mg)	61	61	65	63
Calcium (mg)	61	61	65	63
Iron (mg)	61	61	65	63
Niacin (mg)	61	61	65	63
Niacin Equivalents (mg)	61	61	65	63
Riboflavin (mg)	61	61	65	63
Energy Expenditure (kJ)	61	61	59	62

Supplemental Table S2: Effect of treatment on dietary nutrient intake and energy expenditure as determined weighed food diaries and activity diaries. Completers analysis

	**Almond Diet**		**Nut-Free Diet**		P value	P value	P value
	Pre-treatment	Post-treatment	Pre-treatment	Post-treatment	Group	Time	Group*Time
Energy (kJ)	9127 ± 307	9630 ± 328	8969 ± 302	8777 ± 326	0.135	0.387	0.056
Carbohydrate (g)	229 ± 8	212 ± 8	227 ± 8	226 ± 8	0.564	0.079	0.137
Carbohydrate (%en)	41 ± 5	36 ± 5	41 ± 5	42 ± 5	0.001	0.001	<0.0001
Sugars (g)	111 ± 58	100 ± 58	110 ± 58	93 ± 58	0.510	<0.0001	0.388
Protein (g)	94 ± 3	99 ± 3	94 ± 3	88 ± 3	0.178	0.733	0.008
Protein (%en)	18 ± 2	18 ± 2	18 ± 2	17 ± 2	0.853	0.061	0.134
Fat (g)	86 ± 6	106 ± 6	82 ± 6	80 ± 6	<0.0001	0.001	<0.0001
Fat (%en)	35 ± 4	41 ± 4	34 ± 4	34 ± 4	<0.0001	<0.0001	<0.0001
Monounsaturated fat (g)	32 ± 1	47 ± 1	31 ± 1	29 ± 1	<0.0001	<0.0001	<0.0001
Monounsaturated fat (%en)	13 ± 2	19 ± 2	13 ± 2	12 ± 2	<0.0001	<0.0001	<0.0001
Polyunsaturated fat (g)	13 ± 5	19 ± 5	12 ± 5	10 ± 5	<0.0001	<0.0001	<0.0001
Polyunsaturated fat (%en)	5 ± 0.3	7 ± 0.3	5 ± 0.3	4 ± 0.3	<0.0001	<0.0001	<0.0001
Saturated fat (g)	34 ± 1	31 ± 1	32 ± 1	34 ± 1	0.797	0.710	0.065
Saturated fat (%en)	14 ± 0	12 ± 0	13 ± 0	14 ± 0	0.027	0.422	<0.0001
Alcohol (g)	8 ± 3	8 ± 4	10 ± 3	11 ± 4	0.253	0.610	0.407
Fibre (g)	28 ± 1	29 ± 1	27 ± 1	24 ± 1	0.019	0.447	0.003
Alpha tocopherol (mg)	12 ± 2	26 ± 2	11 ± 2	10 ± 2	<0.0001	<0.000	<0.0001
Sodium (mg)	2468 ± 1928	2401 ± 1929	2497 ± 1927	2687 ± 1929	0.283	0.608	0.281
Potassium (mg)	3498 ± 118	3618 ± 117	3417 ± 115	3193 ± 116	0.130	0.316	0.009
Magnesium (mg)	402 ± 26	491 ± 25	388 ± 25	329 ± 25	<0.0001	0.102	<0.0001
Calcium (mg)	1082 ± 155	1103 ± 153	1025 ± 154	914 ± 153	0.056	0.167	0.043
Iron (mg)	12 ± 5	14 ± 5	13 ± 5	11 ± 5	0.279	0.605	<0.0001
Niacin (mg)	24 ± 11	25 ± 11	25 ± 11	23 ± 11	0.602	0.444	0.345
Niacin Equivalents (mg)	43 ± 4	44 ± 4	42 ± 4	40 ± 4	0.275	0.570	0.093
Riboflavin (mg)	2.2 ± 0.6	2.9 ± 0.6	2.3 ± 0.6	2.0 ± 0.6	0.004	0.026	<0.0001
Caffeine (mg)	229 ± 117	211 ± 116	205 ± 116	196 ± 116	0.334	0.310	0.757
Energy Expenditure (kJ)	14333 ± 281	14408 ± 271	14611 ± 279	14211 ± 270	0.632	0.832	0.217

Estimated Marginal Means ± SEM presented from Linear Mixed Model, at baseline (pre-treatment) and 12weeks (post-treatment). Statistical significance p<0.05.

Supplemental Table S3: Effect of Treatment on cognition. Completers analysis

	**Almond Diet**		**Nut-Free Diet**				
	Pre-treatment	Post-treatment	Pre-treatment	Post-treatment	Group (*p*)	Time (*p*)	Group x Time Interaction (*p*)
Attention RT (composite Z-score)[1]	0.007 ± 0.084	0.003 ± 0.080	-0.001 ± 0.084	-0.079 ± 0.079	0.673	0.373	0.419
Attention Accuracy (composite Z-score)[2]	-0.084 ± 0.072	-0.039 ± 0.074	0.075 ± 0.070	0.044 ± 0.073	0.213	0.841	0.255
Working Memory RT (composite Z-score)[3]	0.046 ± 0.069	-0.019 ± 0.071	0.020 ± 0.070	-0.069 ± 0.070	0.667	0.092	0.804
Working Memory Accuracy (composite Z-score)[4]	-0.119 ± 0.082	-0.062 ± 0.089	0.114 ± 0.079	0.080 ± 0.087	0.108	0.692	0.131
Long Term memory Accuracy (composite Z-score)[5]	-0.056 ± 0.63	0.010 ± 0.074	-0.056 ± 0.063	0.115 ± 0.073	0.444	0.002	0.368
Executive Function Accuracy (composite Z-score)[6]	-0.154 ± 0.073	-0.079 ± 0.078	0.080 ± 0.071	0.125 ± 0.077	0.030	0.084	0.660
Simple RT (ms)	348 ± 13	350 ± 12	351 ± 13	345 ± 12	0.938	0.859	0.658
Choice RT -correct responses (%)	94.84 ± 0.76	94.85 ± 0.58	94.55 ± 0.74	94.82 ± 0.57	0.838	0.782	0.798
Choice RT (ms)	489 ± 11	502 ± 11	498 ± 11	486 ± 11	0.794	0.970	0.129
Four choice RT- correct responses (%)	99.67 ± 0.10	99.73 ± 0.13	99.71 ± 0.10	99.49 ± 0.13	0.462	0.403	0.126
Four choice RT (ms)	691 ± 16	682 ± 17	709 ± 16	675 ± 16	0.812	0.008	0.132
Rapid visual information processing -correct responses (%)	32.9 ± 2.5	36.2 ± 2.7	35.6 ± 2.5	38.6 ± 2.7	0.463	0.018	0.915
Rapid visual information processing RT (ms)	540 ± 9	537 ± 8	537 ± 9	539 ± 8	0.967	0.951	0.666
Rapid visual information processing (false alarms)	9.9 ± 1.9	11.7 ± 1.9	11.9 ± 1.8	12.3 ± 1.8	0.622	0.148	0.386
Numeric working memory accuracy (%)	89.41± 1.20	89.88 ± 1.21	91.60 ± 1.18	92.65 ± 1.19	0.095	0.360	0.819
Numeric working memory RT (ms)	1303 ± 43	1292 ± 42	1241 ± 42	1180 ± 41	0.127	0.048	0.172
N-Back correct Reponses (%)	74.17 ± 2.56	73.99 ± 2.88	75.71 ± 2.52	74.59 ± 2.84	0.749	0.732	0.804
N-Back RT (ms)	870 ± 35	892 ± 39	916 ± 34	888 ± 38	0.632	0.898	0.352
Corsi Blocks -span (score)	5.13 ± 0.10	5.19 ± 0.12	5.32 ± 0.10	5.08 ± 0.12	0.770	0.333	0.095
Corsi Blocks RT (ms)	8461 ± 322	7781 ± 297	8780 ± 369	8304 ± 293	0.297	0.025	0.689
Peg and Ball planning time (before responding) (ms)	6435 ± 322	6057 ± 292	6007 ± 317	5670 ± 288	0.364	0.018	0.886
Peg and Ball execution time (ms)	16872 ± 671	15908 ± 580	16094 ± 662	14706 ± 571	0.242	0.000	0.632
Peg and Ball Errors (score)	4.57 ± 0.47	4.00 ± 0.48	4.16 ± 0.46	3.18 ± 0.47	0.231	0.069	0.632
Immediate Word Recall- correct responses(score)	5.33 ± 0.25	5.60 ± 0.25	5.72 ± 0.24	6.47 ± 0.24	0.040	0.004	0.159
Delayed Word Recall- correct responses (score)	3.58 ± 0.25	4.01 ± 0.27	3.98 ± 0.25	4.36 ± 0.26	0.236	0.023	0.880
Word Recognition- correct responses (%)	80.05 ± 1.18	81.61 ± 1.12	78.02 ± 1.17	81.69 ± 1.10	0.479	0.003	0.240
Word Recognition RT (ms)	1251 ± 40	1268 ± 43	1315 ± 39	1338 ± 42	0.201	0.441	0.913
Picture Recognition- correct responses (%)	97.25 ± 0.45	96.63 ± 0.50	96.43 ± 0.044	96.73 ± 0.49	0.533	0.633	0.177
Picture Recognition RT (ms)	948 ± 28	940 ± 27	996 ± 28	988 ± 27	0.197	0.528	0.998
Stroop Congruent accuracy (%)	99.08 ± 0.33	99.02 ± 0.29	99.23 ± 0.32	98.82 ± 0.28	0.927	0.389	0.525
Stroop Incongruent accuracy (%)	94.75 ± 0.98	95.09 ± 0.94	97.14 ± 0.96	97.75 ± 0.93	0.043	0.391	0.814
Stroop Congruent RT (ms)	1168 ± 41	1118 ± 38	1130 ± 40	1084 ± 37	0.470	0.062	0.937
Stroop Incongruent RT (ms)	1244 ± 46	1259 ± 43	1262 ± 45	1230 ± 42	0.923	0.704	0.296

Serial 3 (number of responses)	20.5 ± 1.3	20.3 ± 1.4	24.1 ± 1.3	24.1 ± 1.3	0.047	0.781	0.815
Serial 3 accuracy (%)	18.3 ± 1.4	17.8 ± 1.4	21.8 ± 1.4	21.1 ± 1.4	0.077	0.225	0.899
Serial 7 (number of responses)	14.7 ± 1.2	15.3 ± 1.2	18.7 ± 1.1	18.4 ± 1.2	0.032	0.687	0.323
Serial 7 accuracy (%)	11.5 ± 1.2	12.1 ± 1.3	15.7 ± 1.2	15.2 ± 1.2	0.030	0.935	0.266

Estimated Marginal Means ± SEM presented from Linear Mixed Model (including age, gender and BMI as covariates), at baseline (pre-treatment) and 12weeks (post-treatment). Statistical significance $p<0.05$. RT=reaction time, ms=milliseconds.

[1]Composite score for Attention -Reaction Time (RT) = ($_z$Simple RT + $_z$Choice RT + $_z$Four Choice RT + $_z$Rapid Visual Information Processing)/4

[2]Composite score for Attention -Accuracy= ($_z$Choice RT + $_z$Four Choice RT + $_z$Rapid Visual Information Processing + $_z$Serial subtraction 3 + $_z$Serial subtraction 7)/5

[3]Composite score for Working Memory -RT= ($_z$Numeric Working Memory + $_z$N-back + $_z$Corsi blocks + $_z$Rapid Visual Information Processing)/4

[4]Composite score for Working Memory -Accuracy= ($_z$Numeric Working Memory + $_z$N-back + $_z$Corsi blocks + $_z$Serial subtraction 3 + $_z$Serial subtraction 7 + $_z$Rapid Visual Information Processing)/6

[5]Composite score for Long Term Memory= ($_z$Delayed Word Recall + zDelayed Word Recognition + $_z$Delayed Picture Recognition + $_z$Verbal Fluency + $_z$Verbal Fluency Exclusion)/5

[6]Composite score for Executive Function= ($_z$Stroop Congruent correct + $_z$Stroop Incongruent correct + $_z$Verbal Fluency + $_z$Verbal Fluency Exclusion + $_z$Serial subtraction 3 + $_z$Serial subtraction 7 + $_z$Peg and Ball (accuracy=negatively scored errors))/7

Supplemental Table S4: Effect of Treatment on mood. Completers analysis

	**Almond Diet**		**Nut-Free Diet**				
	Pre-treatment	Post-treatment	Pre-treatment	Post-treatment	Group (*p*)	Time (*p*)	Group x Time Interaction (*p*)
Profile of Mood States							
Total mood disturbance	1.36 ± 2.01	1.42 ± 2.24	-3.62 ± 2.0	-2.12 ± 2.2	0.106	0.591	0.606
Tension	4.87 ± 0.57	4.37 ± 0.44	4.08 ± 0.57	3.62 ± 0.43	0.219	0.192	0.958
Depression	2.22 ± 0.43	2.50 ± 0.51	1.15 ± 0.43	1.42 ± 0.50	0.052	0.463	0.983
Anger	1.52 ± 0.37	1.04 ± 0.34	1.08 ± 0.37	1.03 ± 0.33	0.573	0.390	0.477
Fatigue	4.33 ± 0.43	4.85 ± 0.57	3.38 ± 0.43	3.73 ± 0.56	0.086	0.259	0.820
Confusion	5.35 ± 0.43	5.18 ± 0.43	4.43 ± 0.43	4.19 ± 0.42	0.078	0.448	0.912
Vigour	16.90 ± 0.75	16.53 ± 0.79	17.77 ± 0.74	16.11 ± 0.78	0.818	0.033	0.174
Bond-Lader Visual Analogue Scale							
Alert	55.12 ± 2.00	50.08 ± 2.04	61.35 ± 1.96	60.93 ± 2.00	0.122	0.118	0.053
Content	67.22 ± 1.88	70.08 ± 2.06	71.99 ± 1.86	71.84 ± 2.03	0.201	0.228	0.181
Calm	50.20 ± 2.33	54.28 ± 2.17	53.34 ± 2.31	56.69 ± 2.13	0.326	0.011	0.800
Karolinska Sleepiness Score	4.15 ± 0.19	4.20 ± 0.20	4.08 ± 0.13	4.16 ± 0.19	0.808	0.561	0.879

Estimated Marginal Means ± SEM presented from Linear Mixed Model*, at baseline (pre-treatment) and 12weeks (post-treatment). Statistical significance p<0.05

* Statistical model for Profile of Mood States variables included age, gender, body mass index (BMI) and Bond-Lader Alertness as covariates.

* Statistical model for Bond-Lader variables included age, gender, BMI and Karolinska Sleepiness score as covariates.

* Statistical model for Karolinska Sleepiness Score included age, gender and BMI

Supplemental Table S5: Effect of Treatment on Cardiometabolic Parameters. Completers analysis

	**Almond Diet**		**Nut-Free Diet**				
	Pre-treatment	Post-treatment	Pre-treatment	Post-treatment	Group (*p*)	Time (*p*)	Group x Time Interaction (*p*)
Total cholesterol (mmol/L)	5.12 ± 0.13	4.92 ± 0.12	5.25 ± 0.13	5.21 ± 0.11	0.199	0.032	0.154
HDL cholesterol (mmol/L)	1.43 ± 0.05	1.45 ± 0.05	1.47 ± 0.05	1.49 ± 0.05	0.558	0.344	0.985
LDL cholesterol (mmol/L)	3.08 ± 0.12	2.94 ± 0.11	3.27 ± 0.12	3.19 ± 0.10	0.153	0.031	0.502
Triglycerides (mmol/L)	1.30 ± 0.07	1.16 ± 0.06	1.13 ± 0.06	1.16 ± 0.06	0.282	0.090	0.004
Ratio Total chol:HDL	3.87 ± 0.14	3.58 ± 0.12	3.72 ± 0.14	3.62 ± 0.12	0.764	<0.0001	0.074
Systolic Blood Pressure (mm/Hg)	133 ± 2	128 ± 2	133 ± 2	132 ± 2	0.403	0.001	0.052
Diastolic Blood Pressure (mm/Hg)	76 ± 1	75 ± 1	76 ± 1	76 ± 1	0.653	0.017	0.177
Glucose (mmol/L)	5.6 ± 0.1	5.6 ± 0.1	5.6 ± 0.1	5.5 ± 0.1	0.665	0.294	0.395
Insulin (mU)	6.98 ± 0.50	7.27 ± 0.49	7.37 ± 0.49	7.06 ± 0.48	0.889	0.959	0.206
Small Arterial Compliance (ml/mmHg x 100)	5.0 ± 0.4	5.2 ± 0.4	5.0 ± 0.3	5.3 ± 0.4	0.856	0.304	0.825
Large Arterial Compliance (ml/mmHg x 10)	15.1 ± 0.5	16.2 ± 0.5	16.2 ± 0.5	16.4 ± 0.5	0.311	0.063	0.214
Intracellular Adhesion Molecule (ng)	168 ± 11	184 ± 20	168 ± 11	152 ± 19	0.406	0.993	0.162
Vascular Cell Adhesion Molecule (ng)	1123 ± 29	1148 ± 32	1056 ± 29	1050 ± 31	0.045	0.993	0.162
HOMA2-IR	0.94 ± 0.07	0.98 ± 0.07	0.98 ± 0.07	0.94 ± 0.06	0.954	0.920	0.192
HOMA2-%B	70.2 ± 3.9	71.6 ± 3.2	71.4 ± 3.3	71.2 ± 3.1	0.928	0.714	0.632
HOMA2-%S	137.5 ± 9.7	134.8 ± 8.4	141.5 ± 9.5	138.2 ± 8.2	0.753	0.570	0.957
Alpha tocopherol (µg/mL)	4.85 ± 0.26	4.86 ± 0.21	4.69 ± 0.25	4.53 ± 0.21	0.435	0.526	0.481
logCRP	0.327 ± 0.054	0.346 ± 0.058	0.359 ± 0.053	0.304 ± 0.057	0.941	0.588	0.260
Weight (kg) *	84.09 ± 1.48	84.38 ± 1.46	84.22 ± 1.45	84.16 ± 1.44	0.983	0.370	0.151
BMI (kg/m^2) *	30.24 ± 0.45	30.36 ± 0.45	30.78 ± 0.45	30.36 ± 0.45	0.922	0.284	0.127
Waist Circumference (cm) *	100.9 ± 1.2	101.1 ± 1.2	101.6 ± 1.2	101.4 ± 1.1	0.754	0.818	0.331
Body fat (%) *	35.7 ± 0.7	35.7 ± 0.7	35.4 ± 0.7	35.3 ± 0.7	0.683	0.507	0.850

Estimated Marginal Means ± SEM presented from Linear Mixed Model (including age, gender and BMI as covariates (*statistical model does not include BMI as covariate)), at baseline (pre-treatment) and 12weeks (post-treatment). Statistical significance p<0.05. CRP was logged transformed to account for non-normally distributed data.
